# Editorial for the Special Issue ‘Molecular Mechanisms of Leukemia’

**DOI:** 10.3390/cimb47010014

**Published:** 2024-12-30

**Authors:** Jungeun An, Myunggon Ko

**Affiliations:** 1 Department of Life Sciences, Jeonbuk National University, Jeonju 54896, Republic of Korea; jan@jbnu.ac.kr; 2 Department of Biological Sciences, Ulsan National Institute of Science and Technology, Ulsan 44919, Republic of Korea

Leukemia encompasses a diverse and intricate group of hematological malignancies that arise from hematopoietic stem and progenitors (HSPCs) in the bone marrow. HSPCs occupy the pinnacle of the hematopoietic differentiation hierarchy, giving rise to progressively specialized progenitors and ultimately all blood and immune cell lineages [1]. Maintaining the delicate balance between self-renewal and differentiation of HSPCs is vital for sustaining hematopoietic homeostasis [2,3]. However, this equilibrium is frequently disrupted by a combination of endogenous or environmental stresses, including genetic mutations, epigenetic alterations, and dysregulated interactions within the bone marrow microenvironment [4,5]. Such perturbations, which accumulate with age, can give rise to clonal hematopoiesis, a condition where pre-leukemic HSPC clones gain a competitive advantage, impair normal differentiation processes, and undergo progressive expansion [6,7]. The accrual of additional genetic and epigenetic abnormalities increases the risk of malignant transformation, resulting in the amplification of dysfunctional leukemic cells that infiltrate bone marrow and other hematopoietic organs [8,9]. Therefore, elucidating the fundamental molecular mechanisms underlying leukemogenesis is essential for the development of effective and potentially curative therapies. Despite significant advances in understanding leukemia pathogenesis over recent decades, unfortunately, many critical questions remain unanswered. Addressing these gaps necessitates continued investigation to uncover deeper mechanistic insights and to design innovative therapeutic strategies capable of overcoming the multifaceted challenges posed by leukemia.

This Special Issue, titled ‘Molecular Mechanisms of Leukemia’, aims to provide a platform for cutting-edge research that deepens our understanding of both biological underpinnings and translational applications of leukemia studies. By integrating findings from genetic, epigenetic, metabolic, and microenvironmental research, this collection illuminates the complex pathways driving leukemogenesis and highlights new opportunities for refining diagnostic and therapeutic strategies.

The research articles featured in this Special Issue explore a diverse but interconnected range of topics related to leukemia biology, pathogenesis, and therapeutic resistance. Lin et al. investigate the potential of hesperetin, a naturally occurring flavonoid derived from citrus fruits, in leukemia therapy [10]. Their findings demonstrate that hesperetin induces autophagy and apoptosis in leukemia cells through the AMPK/Akt/mTOR signaling pathway. This positions it as a promising adjuvant candidate for combatting drug resistance and enhancing treatment efficacy by targeting specific metabolic and signaling pathways. Elyamany et al. examine the dysregulation of Wnt/β-catenin signaling in elderly acute myeloid leukemia (AML) patients, demonstrating that suppression of Wnt/β-catenin inhibitors, such as SFRP and DKK family genes, contributes hyperactive signaling and poor clinical outcomes [11]. Their study highlights the potential of reactivating Wnt/β-catenin inhibitors as a targeted therapeutic strategy specifically tailored to address unique challenges faced by elderly AML patients.

Moawadh et al. provide critical insights into the role of genetic polymorphism in apoptosis-related genes (*Bcl-2*/*Bax*) and pro-inflammatory cytokines (*TNF-α*/*IL-8*) in the context of myeloproliferative neoplasms (MPNs) [12]. Their research identifies significant associations between specific single-nucleotide polymorphisms (SNPs) and an elevated risk of disease, emphasizing the potential of these genetic markers as valuable tools for diagnostic and prognostic applications in MPN management. Singh et al. investigate the relationship between miRNAs, cellular metabolism, and chemotherapy response, focusing on the role of hsa-miR-203a-5p in restoring imatinib sensitivity in resistant chronic myeloid leukemia (CML) cells by modulating GSH metabolism [13]. This study highlights the pivotal role of metabolic pathways in chemoresistance and underscores the promise of miRNA-based therapeutic strategies for overcoming drug resistance in leukemia. Finally, Szmajda-Krygier et al. explore the expression patterns of *RUNX1* and *RUNX3* genes in adult acute lymphoblastic leukemia (ALL) patients [14]. Their findings reveal that RUNX1 is associated with aggressive disease progression, while RUNX3 dysregulation is linked to Philadelphia chromosome-positive cases. These results offer novel insights into the role of RUNX family genes as potential biomarkers and therapeutic targets. Collectively, these studies highlight the multifactorial nature of leukemogenesis, underscoring the necessity of comprehensive approaches that integrate molecular, cellular, and systemic perspectives to unravel the complexity of leukemia biology. Moreover, the findings emphasize the imperative of translating molecular discoveries into clinically viable strategies to address therapeutic resistance and improve patient outcomes.

In recent years, breakthroughs in high-throughput genomic technologies and sophisticated models of hematopoiesis have profoundly enhanced the molecular characterization of leukemia. Despite these advances, translating molecular insights into durable clinical outcomes remains a significant challenge. The inherent inter- and intra-patient heterogeneity of leukemia complicates the development of universally effective therapies, while the dynamic interactions between leukemic cells and their surrounding microenvironment continue to hinder treatment efficacy. Addressing these barriers will require future research to prioritize the integration of multi-omics platforms, cutting-edge imaging modalities, and innovative therapeutic approaches. Collaborative efforts among researchers, clinicians, and industry partners will be indispensable for transforming these advancements into meaningful clinical applications that benefit patients.

In conclusion, the Special Issue ‘Molecular Mechanisms of Leukemia’ highlights the importance of bridging basic and translational research to address the unmet challenges of leukemia. We extend heartfelt gratitude to all authors for their invaluable contributions, the reviewers for their insightful feedback, and the editorial team for their steadfast support. It is our hope that this collection serves as a catalyst for continued exploration and innovation in the field, advancing the shared goal of combating leukemia and enhancing patient care worldwide.
